# Pneumatocele Development after Deceased-Donor Liver Transplantation for Multiple Hepatic Cysts due to Autosomal Dominant Polycystic Kidney Disease: A Case Report

**DOI:** 10.70352/scrj.cr.24-0005

**Published:** 2025-03-25

**Authors:** Atsushi Yoshiyama, Mitsuaki Kawashima, Sodai Nagata, Takahito Fukushima, Koji Aze, Yue Cong, Keita Nakao, Masayuki Nakao, Gouji Toyokawa, Chihiro Konoeda, Kiyoshi Hasegawa, Masaaki Sato

**Affiliations:** 1 Department of Thoracic Surgery, The University of Tokyo Hospital, Tokyo, Japan; 2 Hepato-Biliary-Pancreatic Surgery Division, Department of Surgery, The University of Tokyo Hospital, Tokyo, Japan

**Keywords:** pneumatocele, pulmonary cyst, liver transplantation, atelectasis, positive-pressure ventilation

## Abstract

**INTRODUCTION:**

A pneumatocele is a cystic change in the lung that can develop as a sequelae of infection, inflammation, positive-pressure ventilation, thoracic trauma, and rarely after lung resection. Pneumatocele development triggered by an extrathoracic etiology is rare. Herein, we report a case of a pneumatocele that developed after a deceased-donor liver transplantation.

**CASE PRESENTATION:**

A 57-year-old woman with a diagnosis of autosomal dominant polycystic kidney disease underwent deceased-donor liver transplantation for polycystic liver disease. She did not have any background lung disease, although her right lower lobe was mostly atelectatic due to a remarkably elevated diaphragm. The liver transplant itself was uneventful. A small hole was made in the right diaphragm during the dissection of the liver, but it was successfully repaired without any injury to the lung. On postoperative day 1, the chest radiograph revealed a round hypertranslucency on the right side, which was initially considered subphrenic air retention, and no further evaluation was made at that time. Given that the hypertranslucency persisted, follow-up computed tomography was performed on postoperative day 18, and revealed an air–fluid level above the diaphragm in the right thoracic cavity. Thoracoscopic investigation revealed an intrathoracic hematoma within a pneumatocele in the right lower lobe, which was not detected in the pretransplant computed tomography. The hematoma was removed, and the pneumatocele was resected.

**CONCLUSIONS:**

We experienced a case of a pneumatocele that developed after deceased-donor liver transplantation for multiple hepatic cysts due to autosomal dominant polycystic kidney disease. Although the mechanisms are speculative, the pneumatocele might have been triggered by the sudden alleviation of the elevated diaphragm and reinflation of the atelectatic lung.

## Abbreviations

ADPKD
: autosomal dominant polycystic kidney disease
CT
: computed tomography
DDLT
: deceased-donor liver transplantation
PCLD
: polycystic liver disease
POD
: postoperative day

## INTRODUCTION

Pneumatocele is a cystic change in the lung that can develop as a sequelae of infection, inflammation, positive-pressure ventilation, thoracic trauma, and rarely after lung resection.^1–3)^ The development of a pneumatocele is rarely triggered by extrathoracic etiology. Herein, we report a case of a newly developed pneumatocele accompanied by hemorrhage after deceased-donor liver transplantation (DDLT).

## CASE PRESENTATION

**Fig. 1:**
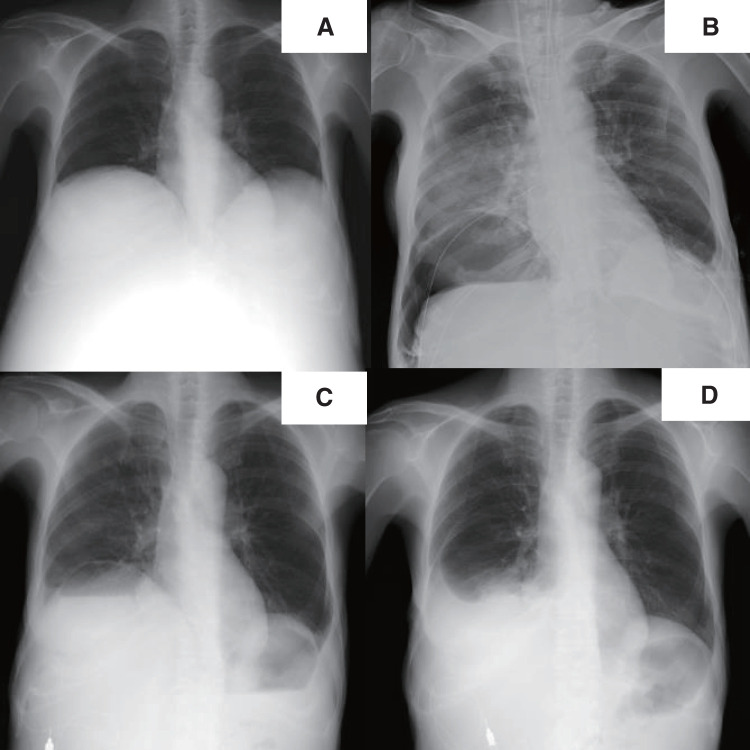
X-ray imaging. Before liver transplantation, the right diaphragm was significantly elevated (**A**). From the first day after transplantation, a cystic shadow was observed in the right lower lung field (**B**), and an air–fluid level was observed within the cyst before the pneumatocele operation (**C**). There was no cystic shadow on the chest X-ray image at the time of discharge (**D**).

**Fig. 2:**
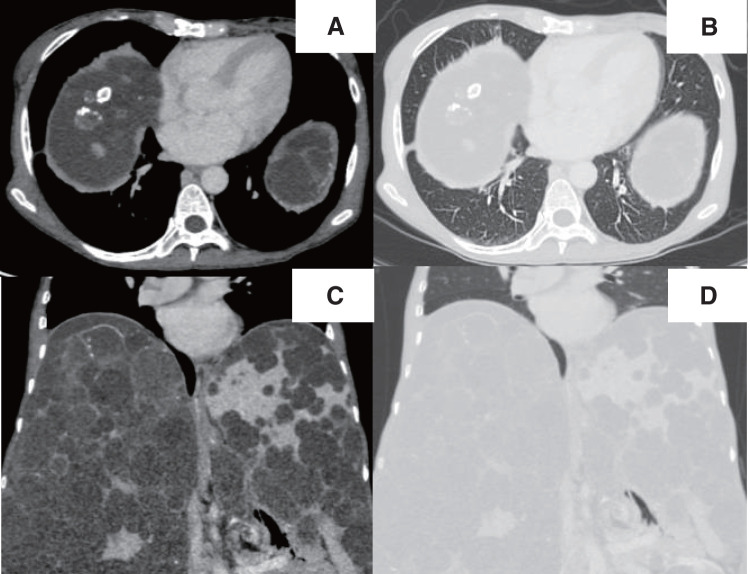
Contrast-enhanced CT findings. Before liver transplantation, CT revealed that the right diaphragm was being pushed up by multiple hepatic cysts. Axial view using a soft tissue window setting (**A**) and lung window setting (**B**), and coronal view using a soft tissue window setting (**C**) and lung window setting (**D**). CT, computed tomography

A 57-year-old female patient with a diagnosis of autosomal dominant polycystic kidney disease underwent DDLT for polycystic liver disease (PCLD). She did not have any background lung disease or smoking history, as shown in her clear preoperative chest radiograph (**Fig. 1A**). Her right diaphragm was severely elevated due to PCLD, which caused persistent atelectasis in the right lower lobe (**Fig. 2A**–**2D**). DDLT was performed uneventfully. A small hole was made on the right diaphragm, which was repaired with a multifilament suture, and a thoracic drain was placed. Although the patient required reoperation on postoperative day 2 (POD 2) due to a persistent bile leakage, she recovered well without further major complications.

**Fig. 3:**
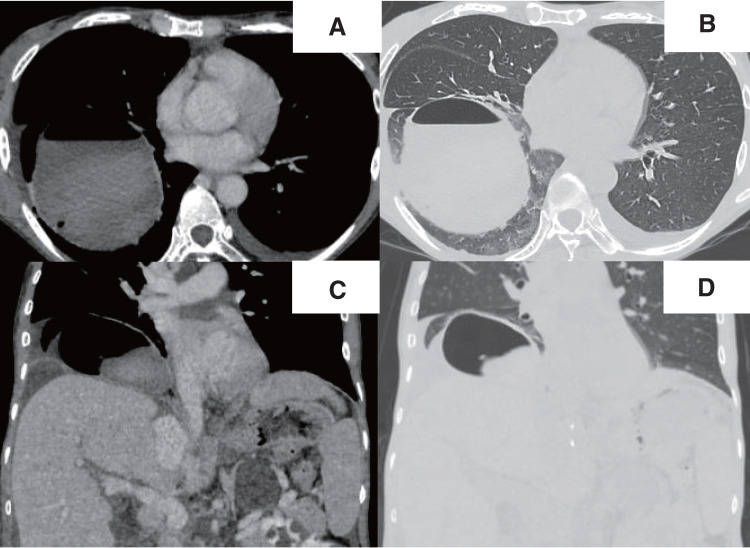
Contrast-enhanced CT images taken just before thoracoscopic surgery, and a large cystic lesion with an air–fluid level was observed adjacent to the right diaphragm. Axial view using a soft tissue window setting (**A**) and lung window setting (**B**), and coronal view using a soft tissue window setting (**C**) and lung window setting (**D**). CT, computed tomography

A chest radiograph revealed a round hypertranslucency with air–fluid levels on the right side since POD 1 (**Fig. 1B** and **1C**), and no further evaluation was made at that time. However, because the hypertranslucency persisted, follow-up computed tomography (CT) was performed on POD 18, and revealed an air–fluid level above the diaphragm in the right thoracic cavity (**Fig. 3A**–**3D**). The patient was initially considered to have an intrathoracic hematoma, and thoracoscopic evacuation of the hematoma was planned.

Regarding the immunosuppressants for liver transplantation, tacrolimus and methylprednisolone were initiated intravenously immediately after the transplantation without any induction therapy. No mycophenolate mofetil was used. Tacrolimus was maintained with a target level of 12–16 ng/mL, and methylprednisolone was tapered according to the institutional protocol. Both tacrolimus and methylprednisolone were switched to oral administration when ready. The liver graft was functioning well with normal transaminases and bilirubin levels, and there were no signs of rejection throughout the course. On the day of the surgery for the pneumatocele (POD 19), the doses of tacrolimus and methylprednisolone were 18 and 16 mg/day, respectively.

**Fig. 4:**
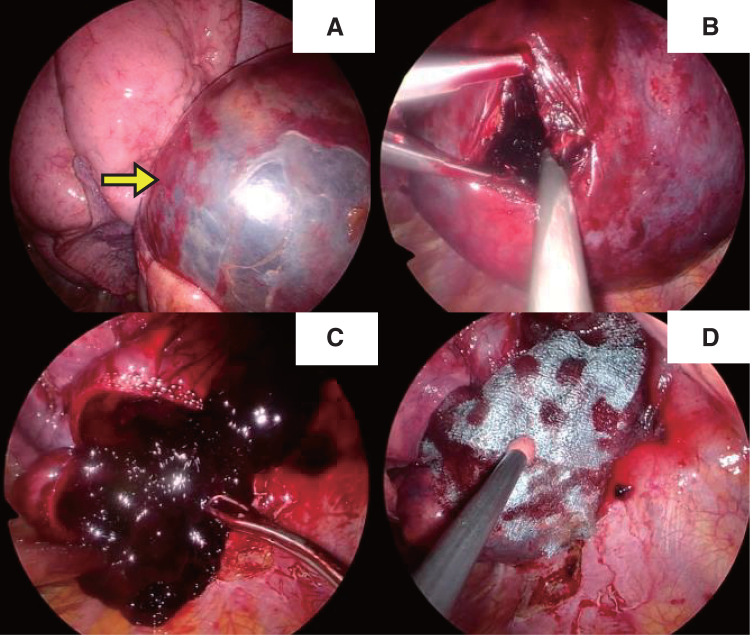
Intraoperative findings. A large pulmonary cyst (arrow) was identified in the right lower lobe (**A**). The cyst wall was incised (**B**). After clot evacuation, wedge resection was performed (**C**). The suture lines were covered with a polyglycolic acid sheet and fibrin glue (**D**).

Contrary to our initial expectation, thoracoscopic investigation revealed no intrathoracic hemorrhage; instead, there was a large cystic change inside the lung parenchyma, which was thought to be a pneumatocele that had formed at the basal area of the right lower lobe (**Fig. 4A**). Surgical resection was preferred given the risk of pneumatocele infection due to intra-cystic hematoma and immunosuppression for liver transplantation. Wedge resection was satisfactory to preserve as much remaining lung parenchyma as possible. From a different perspective, wedge resection was a better way to lower the risk of postoperative empyema, which is higher in lobectomy or segmentectomy. However, simple wedge resection was impractical considering the thickness of the lung parenchyma adjacent to the pneumatocele. It was thought that if the intra-cystic hematoma was evacuated, the thickness of the adjacent lung parenchyma would become thinner and would allow wedge resection with a stapling device. Therefore, the pneumatocele was incised, and the intra-cystic clot was removed to reduce its size (**Fig. 4B** and **4C**). Wedge resection was subsequently performed using a stapling device. The staple line was reinforced with 4-0 polypropylene sutures, a polyglycolic acid sheet (Gunze, Osaka, Japan), and fibrin glue (KM Biologics, Tokyo, Japan) (**Fig. 4D**). The postoperative course of the patient was uneventful, and she was discharged 18days after resecting the pneumatocele (**Fig. 1D**).

Histopathological examination revealed a cyst-free wall, which consisted of visceral pleura with fibrosis and microvascular proliferation. The parenchymal side of the cyst wall was fibrotic and organized with hemorrhage and bronchiolar dilation.

## DISCUSSION

A pneumatocele is a rare condition that can develop as sequelae of infection, inflammation, positive-pressure ventilation, thoracic trauma, and rarely after lung resection.^1–3)^ There are presumably 2 different mechanisms for pneumatocele development. One is positive-pressure driven, and the other is negative-pressure driven. In the former, excessive air entry caused by a bronchial check-valve or mechanical ventilation forcibly destroys alveolar spaces and the lung parenchyma.^2,4)^ In the latter, exacerbated transpleural pressure induced by sudden volume changes in the pleural space (e.g., remaining space after lobectomy) vigorously pulls the pleura and detaches subpleural tissue and the lung parenchyma.^5–7)^ Our case seems to fall into the latter category because the sudden alleviation of diaphragm elevation must have induced negative pressure in the thorax.

The diaphragmatic surface of the lung is reportedly the most common location of pneumatocele development after lobectomy.^5–7)^ The diaphragmatic surface is potentially vulnerable to volume or pressure changes after lobectomy because this space is relatively free from the chest wall. Similarly, in our case, the alleviation of the diaphragm elevation produced extra space above the diaphragm and presumably affected the diaphragmatic surface of the lower lobe.

From a different perspective, another case report describes pneumatocele development along the aortic notch of the left upper lobe after left lower lobectomy.^8)^ The aortic notch of the lung is somewhat atelectatic because of compression by the descending aorta, which can be liberated after lobectomy. Based on that report, it is reasonable to hypothesize that pneumatocele development may be associated with long-lasting atelectasis. In our present case, the patient’s lower lobe had been atelectatic for a long time because of the severely elevated diaphragm. We speculate that the long-lasting atelectasis in our patient was reinflated and formed a pneumatocele after the alleviation of the elevated diaphragm.

It is likely that experiencing pneumatocele formation triggered by DDLT for PCLD is uncommon. However, surprisingly, almost identical cases have been reported in the literature, in which a 46-year-old woman developed a pneumatocele after orthotopic liver transplantation and cadaveric kidney transplantation for polycystic liver and kidney disease.^4)^ Her case was complicated by septic shock due to an infected pneumatocele, which required surgical resection. Although it is difficult to prove, taken together with our case, there seems to be a legitimate rationale behind pneumatocele development after liver transplantation for PCLD. We speculate that the pathogenesis of these 2 cases involves the following steps: (1) The enlarged liver due to PCLD remarkably elevated the diaphragm, causing persistent atelectasis in the lung parenchyma. (2) Liver transplantation suddenly alleviated the diaphragm elevation, generating some extent of negative pressure, and led to the re-expansion of the atelectatic lung. (3) A sudden re-expansion of the atelectatic lung parenchyma provoked structural damage and formed a pneumatocele with hemorrhage.

The typical appearance of a pneumatocele on CT is a round thin-walled cavitary lesion with air–fluid levels.^9,10)^ In our case, the CT findings were compatible with a pneumatocele (**Figs. 3A**–**3D**), but we could not make a correct preoperative diagnosis. This was largely driven by the incorrect assumption that liver surgery would not cause structural changes in the lung parenchyma unless there was direct injury. Additionally, a small hole in the diaphragm could be a source of unusual air retention in the thoracic cavity. Eventually, we considered that a pneumatocele had formed in our patient, triggered by the remarkable alleviation of the distended diaphragm.

The literature reports conservative management, percutaneous drainage, or surgical resection as potential treatment strategies for pneumatocele.^1–10)^ However, no clear guidelines exist, and treatment decisions must be tailored to the individual patient’s clinical context. In our case, surgical resection is likely the preferred approach, given the high risk of pneumatocele infection due to immunosuppression associated with liver transplantation.^4)^ Of course, there is a risk of postoperative infection after lung resection (i.e., empyema), but it is plausible that the risk of pneumatocele infection outweighed the risk of post-lung resection empyema in this case.

## CONCLUSIONS

We experienced a case of a pneumatocele that developed after DDLT for PCLD. Although speculative, in patients with persistent diaphragmatic elevation, a pneumatocele can occur when the sudden resolution of diaphragmatic elevation allows re-expansion of the long-lasting atelectatic lung.

## ACKNOWLEDGMENTS

We thank Robin James Storer, PhD, from Edanz (https://jp.edanz.com/ac) for editing a draft of this manuscript.

## DECLARATIONS

### Funding

None.

### Authors’ contributions

AY and MK drafted the manuscript.

AY, SN, TF, KA, YC, KN, and MN contributed to patient care and data collection.

All authors critically revised the content and approved the final version.

### Availability of data and materials

The datasets supporting the conclusions of this article are included within the article.

### Ethics approval and consent to participate

Not applicable as a case report is not regarded as a study according to the “Ethical Guidelines for Medical and Health Research Involving Human Subjects” of the Japanese Ministry of Health, Labour and Welfare.

### Consent for publication

Written informed consent was obtained from the patient for the publication of this case report, including her medical data and images.

### Competing interests

The authors declare that they have no competing interests.
